# Aberrant axial mineralization precedes spinal ankylosis: a molecular imaging study in *ank/ank *mice

**DOI:** 10.1186/ar3482

**Published:** 2011-10-12

**Authors:** Facundo Las Heras, Ralph S DaCosta, Kenneth PH Pritzker, Nigil Haroon, George Netchev, Hing Wo Tsui, Basil Chiu, W Mark Erwin, Florence WL Tsui, Robert D Inman

**Affiliations:** 1 Institute of Medical Science, University of Toronto, 1 King's College Circle, Toronto, Ontario, M5S 1A8, Canada; 2 Pathology and Laboratory Medicine, Mount Sinai Hospital, 600 University Avenue, Toronto, Ontario, M5G 1X5, Canada; 3 Ontario Cancer Institute, University Health Network, 610 University Avenue, Toronto, Ontario M5G 2M9, Canada; 4 Department of Laboratory Medicine and Pathobiology, University of Toronto, 1 King's College Circle, Toronto, Ontario, M5S 1A8, Canada; 5 Toronto Western Research Institute, University Health Network, 399 Bathurst Street, Toronto, Ontario M5T 2S8, Canada; 6 Division of Orthopaedic Surgery, University of Toronto, 100 College Street, Toronto, Ontario M5G 1L5, Canada; 7 Department of Immunology, University of Toronto, 1 King's College Circle, Toronto, Ontario M5S 1A8, Canada; 8 Department of Medical Biophysics, University of Toronto, 1 King's College Circle, Toronto, Ontario, M5S 1A8, Canada; 9 Toronto Western Hospital, 399 Bathurst Street, Toronto, Ontario M5T 2S8, Canada

## Abstract

**Introduction:**

The diagnosis of ankylosing spondylitis is made from a combination of clinical features and the presence of radiographic evidence that may be detected only after many years of inflammatory back pain. It is not uncommon to have a diagnosis confirmed 5 to 10 years after the initial onset of symptoms. Development of a more-sensitive molecular imaging technology to detect structural changes in the joints would lead to earlier diagnosis and quantitative tracking of ankylosis progression. Progressive ankylosis (*ank/ank*) mice have a loss of function in the *Ank *gene, which codes for a regulator of PPi transport. In this study, we used these *ank/ank *mutant mice to assess a noninvasive, quantitative measure of joint ankylosis with near-infrared (NIR) molecular imaging *in vivo*.

**Methods:**

Three age groups (8, 12, and 18 weeks) of *ank/ank *(15 mice) and wild-type littermates (12 +/+ mice) were assessed histologically and radiographically. Before imaging, OsteoSense 750 (bisphosphonate pamidronate) was injected i.v. Whole-body images were analyzed by using the multispectral Maestro imaging system.

**Results:**

OsteoSense 750 signals in the paw joints were higher in *ank/ank *mice in all three age groups compared with controls. In the spine, significantly higher OsteoSense 750 signals were detected early, in 8-week-old *ank/ank *mice compared with controls, although minimal radiographic differences were noted at this time point. The molecular imaging changes in the *ank/ank *spine (8 weeks) were supported by histologic changes, including calcium apatite crystals at the edge of the vertebral bodies and new syndesmophyte formation.

**Conclusions:**

Changes in joint pathology of *ank/ank *mice, as evaluated by histologic and radiographic means, are qualitative, but only semiquantitative. In contrast, molecular imaging provides a quantitative assessment. Ankylosis in *ank/ank *mice developed simultaneously in distal and axial joints, contrary to the previous notion that it is a centripetal process. NIR imaging might be feasible for early disease diagnosis and for monitoring disease progression in ankylosing spondylitis.

## Introduction

Outbred homozygous *ank *(*progressive ankylosis*) mice represent a highly informative model for studying the biologic basis of joint ankylosis. In these mice, a single gene mutation alters an inorganic pyrophosphate transporter and is associated with spontaneous joint ankylosis [1]. In a pattern similar to that of ankylosing spondylitis (AS) in humans, the ankylosis in these mice affects peripheral and spinal joints [1]. The central features of AS are spinal inflammation and ankylosis [2]. As spinal structural changes as visualized on radiographs appear relatively late in most AS patients, it is not uncommon for a delay of 5 to 10 years after the initial onset of symptoms before the diagnosis is made. Our colony of *ank/ank *mice exhibits ankylosis without joint inflammation [3]. This unique feature offers an opportunity to analyze ankylosis directly in the mutant mice in the absence of confounding joint inflammation.

Near-infrared (NIR) probes, which generate high signal-to-noise ratios, have the potential for noninvasive quantitative fluorescence imaging in whole animals [4]. OsteoSense 750 (VisEN Medical, Bedford, MA, USA) is an NIR fluorescent bisphosphonate (pamidronate) that targets hydroxyapatite for monitoring skeletal changes [5]. With this NIR probe, bone growth and remodeling can be imaged with high sensitivity and resolution, and mineralization can be quantified.

Previous radiographic studies documented that aberrant mineralization in *ank/ank *joints occurred in a centripetal fashion (that is, from distal to proximal joints [6]), but the mechanisms underlying the temporal and progressive manner of ankylosis remain unclear. In this study, we attempted to define the dynamics of ankylosis progression in *ank/ank *mice by using the more-sensitive and quantitative molecular imaging method, and we obtained a surprising result.

## Materials and methods

### *ank *mice and injection of OsteoSense 750 probe

The *ank/ank *mice (outbred) were generated by crossbreeding heterozygous mice. Wild-type (+/+) littermates were used as controls. For this study, 15 mutant (*ank/ank*) and 12 wild-type mice were evaluated. Three age groups of mice were analyzed (8, 12, and 18 weeks). Twenty-four hours before imaging, OsteoSense 750 (VisEN) was injected into the tail veins at the recommended dose of 2 nmol/150 ml per mouse. All animal procedures were approved by the Institutional Animal Experimentation Committee.

### *In vivo *molecular imaging and analysis

Whole-body and lower-body images of injected mice were acquired by using the Maestro *in vivo *imaging system (CRI Inc., Santa Maria, CA, USA; excitation: 671 to 705 nm; emission: 750 to 950 nm). The Maestro optical system uses a liquid crystal tunable filter (30-nm bandwidth; scanning wavelength range, 500 to 950 nm), a 16-bit high-resolution scientific-grade monochrome imaging sensor and Maestro 1.4.2 software for image acquisition and analysis. The imaging regions with pure autofluorescence spectra were manually selected and subtracted from the mixed fluorescence signal of the image to obtain the OsteoSense 750 fluorescence intensity at each imaging pixel recorded. Mean fluorescence intensities from the selected regions were normalized, measured, and finally, analyzed.

### Radiographic and histologic analyses

After NIR imaging, radiographs of the killed mice were generated via a Faxitron unit (Hewlett Packard Faxitron X-ray system 43855 B; San Diego, CA, USA) by using similar settings for each mouse. For detection of structural differences, high-resolution electronic images of the radiographs were taken under similar conditions and exposures for each pair of mice (*wild-type *versus *ank/ank*) for each age group. For histologic analyses, the fixed joints were decalcified in 10% EDTA. After dehydration through a graded alcohol series, the tissues were embedded in paraffin, sectioned (at 5 mm), and stained with hematoxylin and eosin.

### Statistical analysis

Mann-Whitney *U *tests (SPSS v18.0) were used to compare results from the wild-type versus mutant mice of different age groups. The *P *values of < 0.05 were considered significant.

## Results

### Radiographic changes in *ank/ank *mice were observed earlier and more prominently in paw joints than in the axial skeleton

**Figure 1:**
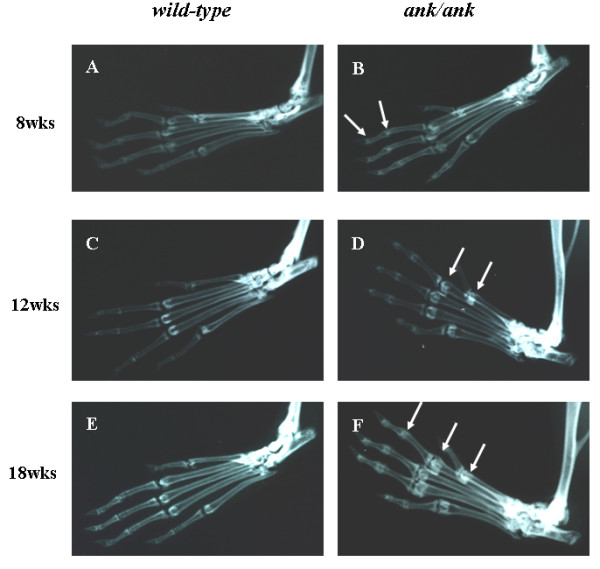
**Radiographs of interphalangeal joints from paws of *wild-type *versus *ank/ank *mice**. We show 8-week-old mice in **(A) **and **(B)**; 12-week-old mice in **(C) **and **(D)**; and 18-week-old mice in **(E) **and **(F)**. White arrows show increased calcification in the interphalangeal joints.

**Figure 2:**
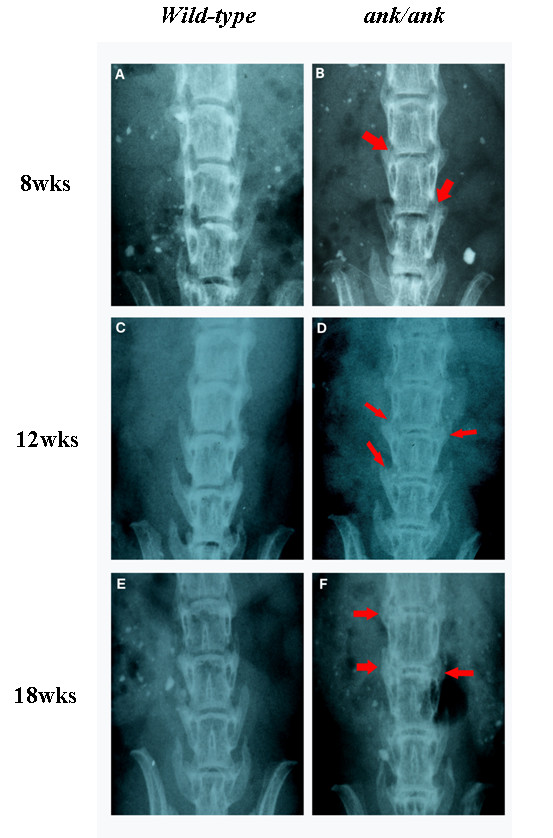
**Radiographs of spines from *wild-type *versus *ank/ank *mice**. Eight-week-old mice appear in **(A) **and **(B)**; 12-week-old mice in **(C) **and **(D)**; and 18-week-old mice in **(E) **and **(F)**. Red arrows in **(B) **show spurs between the vertebral bodies of 8-week-old *ank/ank *mice. Red arrows in **(D) **show early syndesmophytes at the edges of the vertebra from 12-week-old *ank/ank *mice. Red arrows in **(F) **show extensive marginal syndesmophyte formation between vertebral bodies from 18-week-old *ank/ank *mice.

Our radiographic results of the *ank/ank *mice were comparable to those from previous reports [6]. We focused mainly on structural changes, in both the peripheral joints and the spine. The 8-week-old *ank/ank *mice showed increased mineralization in the distal interphalangeal joints (Figure 1B; white arrows). From 12 to 18 weeks, joint calcification became more pronounced progressively and extended to more proximal joints (Figure 1D and 1E; white arrows). The differences in the axial skeleton were subtle at 8 weeks, with early syndesmophytes at the vertebral corners of *ank/ank *mice (Figure 2B; red arrows). By 12 weeks, the corner syndesmophytes were well formed in the *ank/ank *vertebra (Figure 2D; red arrows), which progressed to syndesmophytes by 18 weeks (Figure 2E; red arrows).

Radiographic examination of the sacroiliac joints (SIJs) of *ank/ank *mice (8 and 12 weeks) revealed no ankylosis (Additional File 1). The resolution of the radiographs was insufficient for semiquantitative analysis of the radiographs.

### *In vivo *molecular imaging showed higher axial OsteoSense 750 signals in 8-week-old *ank/ank *mice

**Figure 3:**
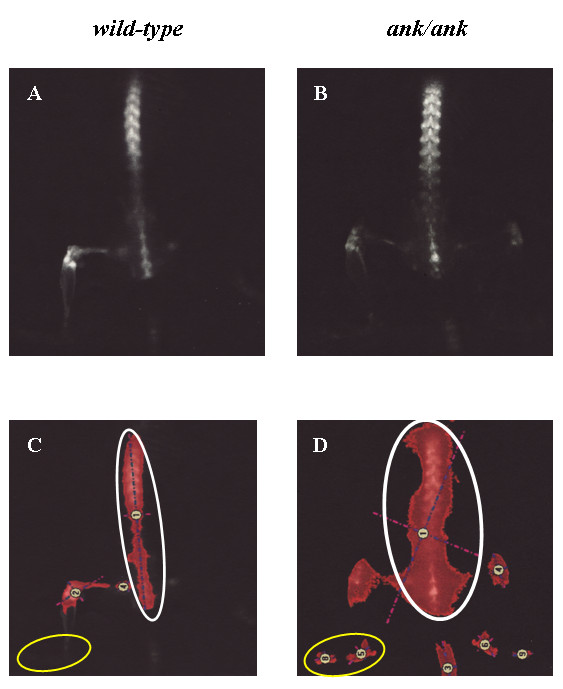
***In vivo *imaging of *wild-type *versus *ank/ank *mice after injection of OsteoSense 750 probes**. **(A, B) **Scans of 12-week-old *wild-type *versus *ank/ank *mice to identify fluorescent signals. For quantification of signals, selected areas for distal paws and spine are circled in yellow and white, respectively **(C, D)**.

**Figure 4:**
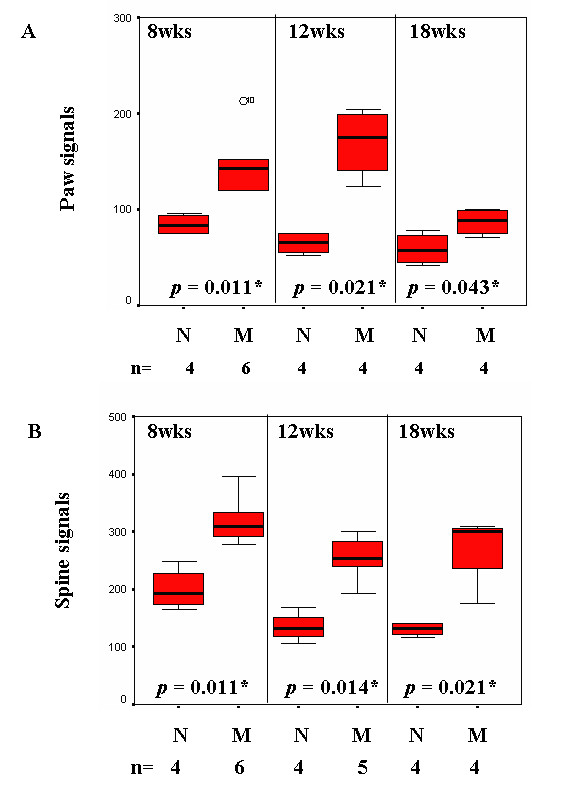
**Boxplots comparing the photon counts emitted by *wild-type *(N) versus *ank/ank *(M) mice at ages 8, 12, and 18 weeks**. N, number of mice used for statistical analysis.

Figure 3 shows the representative biodistribution of OsteoSense 750 signals of *wild-type *versus *ank/ank *mice (12 weeks; Figure 3A and 3B). Areas (in red) were marked for calculation of fluorescence signals (circled). As expected, paw joints of *ank/ank *mice from all three groups showed significantly higher fluorescence signals (expressed in mean photon counts with 95% confidence intervals) than did those of normal (+/+) littermates. The paw signals in *ank/ank *mice were 148 (112.1 to 184.8), 169.5 (111.3 to 227.8), and 87 (64.9 to 109) at 8, 12, and 18 weeks, respectively, compared with 84.4 (67.5 to 101.2), 64.9 (46.1 to 83.6), and 58.9 (31.6 to 86.2) at the corresponding age groups of wild-type littermates (Figure 4A). Interestingly, as early as 8 weeks of age, mean fluorescence signals from the lower spines of *ank/ank *mice were significantly higher than those from the wild-type (+/+) littermates of the same age group. At 8, 12, and 18 weeks, the spinal signals were 319.3 (274.9 to 363.7), 253.7 (177.1 to 330.3), and 271.1 (169.0 to 373.1), respectively, in *ank/ank *versus 200.2 (141.9 to 258.4), 134.7 (93.2 to 176.1), and 130.4 (111.7 to 149.1), respectively, in wild-type mice (Figure 4B). Comparisons of paw and spinal signals between wild-type and *ank/ank *mice in all three age groups showed significant differences (*P *< 0.05 at each age group; Figure 4A and 4B and Table 1).

**Table 1:** Comparisons of fluorescence signals (paw and spine) between *wild-type *(*wt*) and *ank/ank *mice in three different age groups (8, 12, and 18 weeks)

	Paw					Spine				
		*n*	Mean signal	95% confidence intervals	*P*		*n*	Mean signal	95% confidence intervals	*P*
*Wt *(8 weeks)		4	84.4	67.5-101.2			4	200.2	141.9-258.4	
*ank/ank *(8 weeks)		6	148.4	112.1-184.8			6	319.3	274.9-363.7	
Mann-Whitney *U *test					0.011^a^					0.011^a^
*wt*(12 weeks)		4	64.9	46.1-83.6			4	134.7	93.2-176.1	
*ank/ank *(12 weeks)		4	169.5	111.3-227.8			5	253.7	177.1-330.3	
Mann-Whitney test					0.021^a^					0.014^a^
*Wt *(18 weeks)		4	58.9	31.6-86.2			4	130.4	111.7-149.1	
*ank/ank *(18 weeks)		4	87.0	64.9-109			4	271.1	169.0-373.1	
Mann-Whitney test					0.043^a^					0.029^a^

^a^Significant differences; *n *= number of mice.

### Spinal *in vivo *molecular imaging results were paralleled by histologic findings

**Figure 5:**
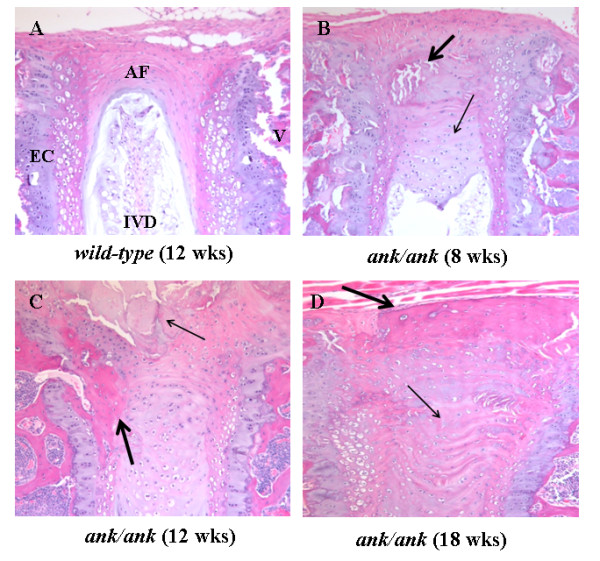
**Histologic examination of representative spine joints from *wild-type *versus *ank/ank *mice**. **(A) **Vertebra from an 8-week-old *wild type *mouse. V, EC, AF, and IVD denote vertebra, endplate cartilage, annulus fibrosus, and intervertebral disc, respectively. **(B) **Vertebra from an 8-week-old *ank/ank *mouse. Thick arrow shows calcium apatite crystals at the edge of the vertebral bodies. Thin arrow shows hypertrophic chondrocyte-like cells replacing the annulus fibrosus and extending into the intervertebral disc. **(C) **Vertebra from a 12-wk-old *ank/ank *mouse. Thin arrow shows a large amount of crystal deposits. Thick arrow shows early syndesmophyte formation at the edge of the vertebral bodies. **(D) **Vertebra from an 18-week-old *ank/ank *mouse. Thick arrow shows ankylosis with fibrous connective tissue adjacent to some incomplete bony bridging. Thin arrow shows the intervertebral disc at this stage, and it was replaced by proliferative fibroblasts and large chondrocytic cells.

**Figure 6:**
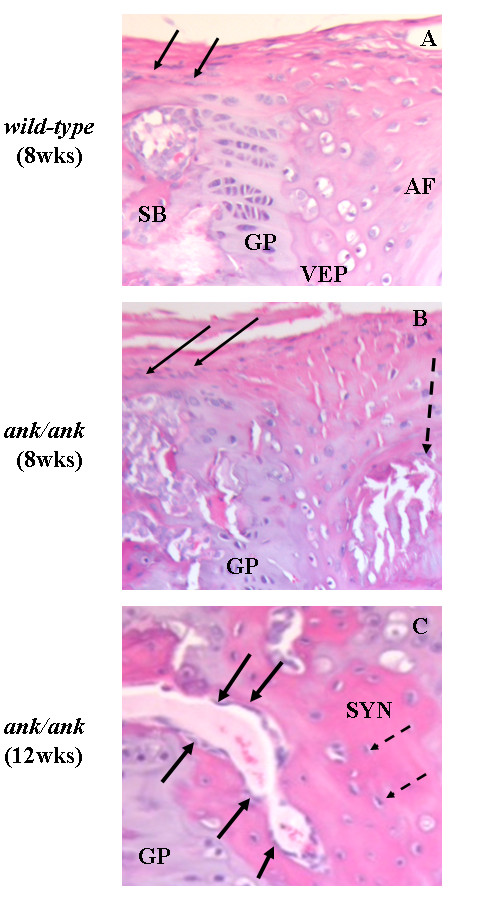
**Higher magnification of spinal sections from 8-week-old *wild-type *(A) versus 8-week- (B), and 12-week-old (C) *ank/ank *mice**. Arrows in **(A) **show a few osteoblasts lining the subchondral bone (SB). GP, VEP, and AF denote growth plate, vertebral end plate, and annulus fibrosus, respectively. Solid arrows in **(B) **show more-prominent osteoblasts in the vertebral body of an 8-week-old *ank/ank *spine. Dashed arrows show calcific deposits. Solid arrows in **(C) **show numerous osteoblasts lining the new bone that was formed in a 12-week-old *ank/ank *mouse. Embedded in the syndesmophyte (SYN) are osteocytes (short dashed arrows).

**Figure 7:**
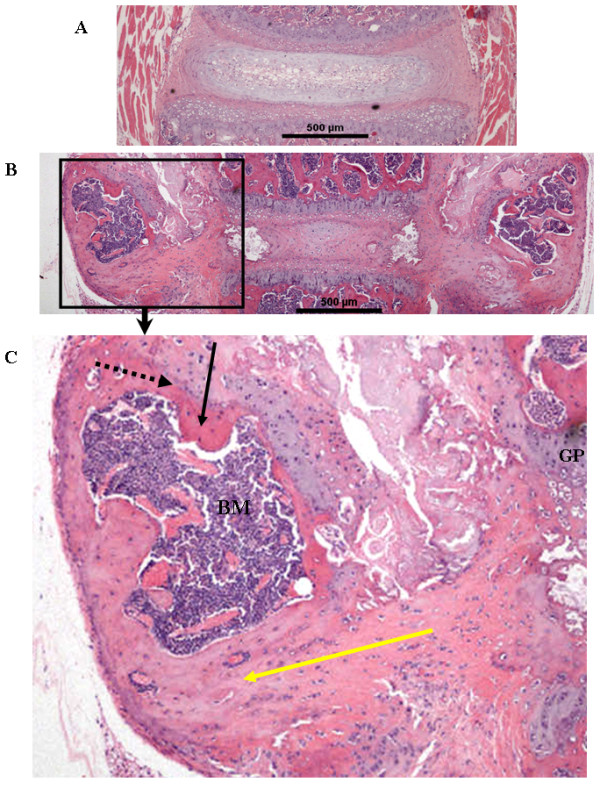
**Comparison of spinal sections from *wild-type *(A) versus *ank/ank *mice across the intervertebral disc (IVD)**. **(B) **A spinal section from an 18-week-old *ank/ank *mouse. Large fusion masses of fibrocartilaginous tissues extend from one vertebra to the next, at the edges of the annulus fibrosus of the IVD. **(C) **A larger view of the marked rectangular area from **(B)**. Within the fusion masses, "bone islands" are complete with bone marrow (BM). These regions demonstrate the classic appearance of the growth plate, such as tidemark areas (dotted black arrow) and organized subchondral layers (black arrow). The connective tissue/fibrous tissue masses seen in the *ank/ank *spine are largely ill defined (yellow arrow).

The histomorphology of the vertebral column was significantly altered in 8-week-old *ank/ank *mice. Calcium apatite crystals were observed at the edges of the vertebral bodies (Figure 5B, thick arrow). Hypertrophic chondrocyte-like cells were replacing the annulus fibrosus (AF) and extending into the intervertebral disc (IVD; Figure 5B, thin arrow). Spinal sections of 12-week-old *ank/ank *mice showed a large amount of crystal deposition (Figure 5C, thin arrow) and early syndesmophyte formation (Figure 5C, thick arrow). The vertebral column of an 18-week-old *ank/ank *mouse showed ankylosis with fibrous connective tissue adjacent to some incomplete bony bridging (Figure 5D, thick arrow). The intervertebral disc at this stage was essentially replaced by a cellular proliferation composed of fibroblasts and large chondrocytic cells (Figure 5D, thin arrow). Higher magnification of the spinal sections showed that in *wild-type *mice, a few osteoblasts line the subchondral bone (Figure 6A, solid arrows). In 8-week *ank/ank *spine, the osteoblasts are more prominent and organized (Figure 6B, solid arrows), and calcific deposits (Figure 6B, dashed arrow) are present. In 12-week *ank/ank *spine, new bone formation (syndesmophyte, SYN; Figure 6C) developed where calcific deposits were located in younger mice. Numerous osteoblasts (solid arrows, Figure 6C) lined the new bone that was formed. Osteocytes are found embedded in the syndesmophyte (short dashed arrows; Figure 6C). In some *ank/ank *(18 weeks) spinal sections, large fusion masses of fibrocartilaginous tissues extend from one vertebra to the next, at the edges of the annulus fibrosus of the IVD (Figure 7). Within the fusion masses, "bone islands" appear, complete with bone marrow. These regions demonstrate the classic appearance of the growth plate, such as tidemark areas and organized subchondral layers (Figure 7, arrow).

## Discussion

In this study, our imaging results revealed a new insight into the axial ankylosis process in the *ank/ank *mice. Previous studies reported that aberrant mineralization in *ank/ank *joints occurred in a centripetal fashion (that is, from distal to proximal joints) [6]. In contrast, by using *in vivo *molecular imaging, we showed that ankylosis in *ank/ank *mice developed simultaneously in distal and axial joints, resulting in complete ankylosis at all skeletal sites by 18 weeks. Our novel molecular imaging finding was corroborated by spinal histologic assessments showing that synoviocytes/fibroblast proliferation and crystal deposits were observed in both interphalangeal (data not shown) and spinal joints as early as 8 weeks in *ank/ank *mice. In addition, our histologic analyses revealed a predominance of large chondrocytic cells at the sites of eventual ankylosis, suggesting that dysregulation of chondrocyte maturation underlies the development of ankylosis in *ank/ank *mice. Although we did not detect overt histologic abnormalities of osteoblasts, we cannot rule out the possibility that *ank/ank *osteoblasts have more activity contributing to excess mineralization in the mutant mice. It has been shown that the Ank protein plays an important role in osteoblastic differentiation [7]. However, osteoblasts (involved in endochondral ossification) might not be the major contributor to syndesmophyte formation. These syndesmophytes appear to be atypical as (a) they usually develop in the same sites where calcific deposition and prominent hypertrophic chondrocytes were located in the spine (enthesis) of younger mutant mice, indicating that they are likely more fibrocartilaginous in nature; (b) the connective tissue/fibrous tissue masses seen in the *ank/ank *spine (Figure 7) are largely ill defined; and (c) no discernible contiguous bone within the syndesmophyte connects one vertebra with the next. These features are in contrast to the typical osteophytes, which contain contiguous bony fusion masses emanating from the vertebral body, complete with bone marrow. To illustrate this latter feature (a typical osteophyte), we include a supplementary figure (Additional File 2) showing the spine from a chondrodystrophic canine (beagle) with diffuse idiopathic skeletal hyperostosis (DISH). The osteophytes affecting the lumbar spine clearly showed contiguous bony fusion (Additional File 2, panel a), and no ill-defined connective tissue/fibrous tissue mass appears (Additional File 2, panel b), which is a prominent feature in *ank/ank *spine.

Published reports have shown that bisphosphonates localize to osteoblastic and osteoclastic surfaces, depending on their side chains and dosages used. It has been documented [8,9] that OsteoSense probe (NIR pamidronate) binds to (a) active osteoblastic surfaces, as shown by co-localization with alkaline phosphatase staining; and (b) osteoclastic surfaces by histology (localized in large osteoclast-like cells in resorption lacunae). However, these results were from very young mice (8-day-old neonates and 7- to 9-week-old mice). With the same NIR (OsteoSense 750) probe, our results showed that in normal mice, paw signals were significantly lower in older mice (for example, *P *= 0.029, comparing 8-week and 12-week *wild-type *mice). It is possible that fewer active surfaces were available in the paws of older mice (12 weeks or older), although no significantly different spine signals were seen between young and older normal mice. As with normal mice, no significant differences were found in spine signals from all three age groups of *ank/ank *mice; but these signals were all higher than those from the normal mice. In contrast, 18-week-old *ank/ank *mice had lower paw signals than did the younger mutant mice (8 versus 18 weeks; *P *= 0.01; 12 versus 18 weeks; *P *= 0.03). It is possible that in the paws, fewer active surfaces were present in the mutant mice by 18 weeks. Osteoblast/mineralization signals represent real-time snapshots of new bone formation. Other factors that might influence Osteosense signals include access of the probe from the vasculature to the mineralized surfaces. In another study [10], although hydroxyapatite (HA) deposits in arteries were detected, "skip" areas within the same vessel were also present. The reason for this phenomenon/heterogeneity remains unclear.

In ankylosing spondylitis, ankylosis is initiated at the sacroiliac joints. However, we did not find any ankylosis in the sacroiliac joints of the *ank/ank *mice (Additional File 1). Radiographs of low resolution might not be the best tool for assessment of ankylosis at the sacroiliac joints of mice. Micro-CT image assessments are required to address this issue. Alternatively, histologic evaluation of sacroiliac joints (not decalcified) after injection of a fluorescent bisphosphonate probe might be able to resolve this issue [11]. Although numerous phenotypic similarities exist between the *ank/ank *mice and AS, it is not unexpected that differences exist as well. Ankylosis in the *ank/ank *mouse is the consequence of a single gene defect. However, AS is a complex disease with multiple risk factors.

In the current study, we used one NIR (OsteoSense 750) probe at one particular fluorescence excitation (750). The capacity exists to use more than one NIR probe simultaneously for molecular imaging. For example, in addition to OsteoSense 750 probe, another NIR probe at fluorescence excitation of 680 (such as MMP) can be injected into the mice for simultaneous imaging of MMP localization and bone remodeling. Other molecular targets could also be imaged by using Alexa Fluor 680 fluorescent dye-conjugated specific antibodies. This has been successfully used for molecular imaging of vascular endothelial growth factor (VEGF) for monitoring cancer treatment by using the Maestro HIS system [12].

We need more-sensitive, objective, and quantitative assessments of joint ankylosis in AS patients. According to the modified New York criteria, the diagnosis of AS requires evidence of radiographic sacroiliitis. Sacroiliac joint radiographs are scored from grade 0 (normal) to grade 4 (ankylosis) [13]. Currently, the cervical and lumbar spines are scored according to the modified Stoke AS Spine Score (mSASSS) [14]. The interpretation of the sacroiliac radiographic assessments is notoriously very difficult. Our observation that higher OsteoSense 750 signals can be detected in 8-week-old *ank/ank *spine in which prominent radiographic abnormalities can yet be detected, suggests that this molecular imaging technology is an alternative to mSASSS for earlier AS diagnosis and the monitoring of disease progression in AS patients.

Among the NIR probes, only indocyanine green (ICG; absorption at 780 nm and emission at 820 nm) is Food and Drug Administration (FDA) approved for clinical use [15]. A recent study demonstrated that ICG-enhanced optical imaging can detect joint inflammation in an antigen-induced arthritic mouse model [16]. Unfortunately, conjugation of ICG to proteins is difficult, as ICG is amphophilic and has few functional groups. In addition, ICG loses its fluorescence when bound to protein. To alleviate this problem, the use of quenching NIR probes (ICG-conjugated monoclonal antibodies) has been proposed [15]. Much further work in this respect is needed. Regarding detection systems, it would be ideal to have a hand-held device [17] to image sacroiliac joints and spine in the rheumatology clinic. The *in vivo *molecular imaging could shorten the time required to diagnose AS. In addition, this technology provides unbiased and high-quality assessments to monitor disease progression as well as the outcome of therapeutic treatments [18,19].

## Conclusions

We showed that molecular imaging is more sensitive than radiographs for the detection of early mineralization changes in the spines of *ank/ank *mice. The molecular imaging findings were supported by spinal histologic changes in the mutant mice. Thus this state-of-the-art imaging technology has the potential to shorten the time required to diagnose AS and to monitor disease progression and outcome of treatments.

## Abbreviations

AF: annulus fibrosus; Ank: progressive ankylosis; AS: ankylosing spondylitis; BM: bone marrow; DISH: diffuse idiopathic skeletal hyperostosis; ICG: indocyanine green; IVD: intervertebral disc; MMP: matrix metalloproteinase; mSASSS: modified Stoke AS Spine Score; NIR: near-infrared; PPi: inorganic pyrophosphate; SIJ: sacroiliac joint; SYN: syndesmophyte; VEGF: vascular endothelial growth factor.

## Competing interests

The authors declare that they have no competing interests.

## Authors' contributions

FLH participated in the design of the study, carried out molecular imaging, analyzed radiographs and pathology, performed statistical analysis, and drafted the manuscript. RSD participated in molecular imaging and analysis of the data. KPHP participated in the design of the study, analysis of pathology, and reviewed the manuscript. NH participated in analysis of the radiographs and reviewed the manuscript. GN participated in molecular imaging. HWT and BC participated in animal breeding, genotyping, and data analysis. WME participated in analysis of the radiographs and pathology, interpretation of the data, and critical review of the manuscript. FWLT and RDI participated in the design of the study, coordinated the study, analyzed and interpreted the data, and revised the manuscript. All authors read and approved the final manuscript for publication.

## Supplementary Material

Additional file 1 **Radiographs comparing the sacroiliac joints (SIJ) from 8- and 12-week-old *wild-type *versus *ank/ank *mice**. No ankylosis was detected. Click here for file

Additional file 2 **An old beagle spine with diffuse idiopathic skeletal hyperostosis (DISH)**. **(a) **A computed tomography (CT) micrograph showing contiguous bony fusion in the lumbar spine, connecting one vertebra to the next. **(b) **Histopathology of a typical osteophyte, which contains a contiguous bony fusion mass emanating from the vertebral body, complete with bone marrow (BM). A prominent feature in *ank/ank *spine (ill-defined connective tissue/fibrous tissue mass) is not observed in the old beagle spine. Click here for file
