# Supplementary figures and images for: Aberrant axial mineralization precedes spinal ankylosis: a molecular imaging study in ank/ank mice
Source: Arthritis Res Ther. 2011 Oct 12;13(5):R163. doi: 10.1186/ar3482 (PMC3308096; doi:10.1186/ar3482)

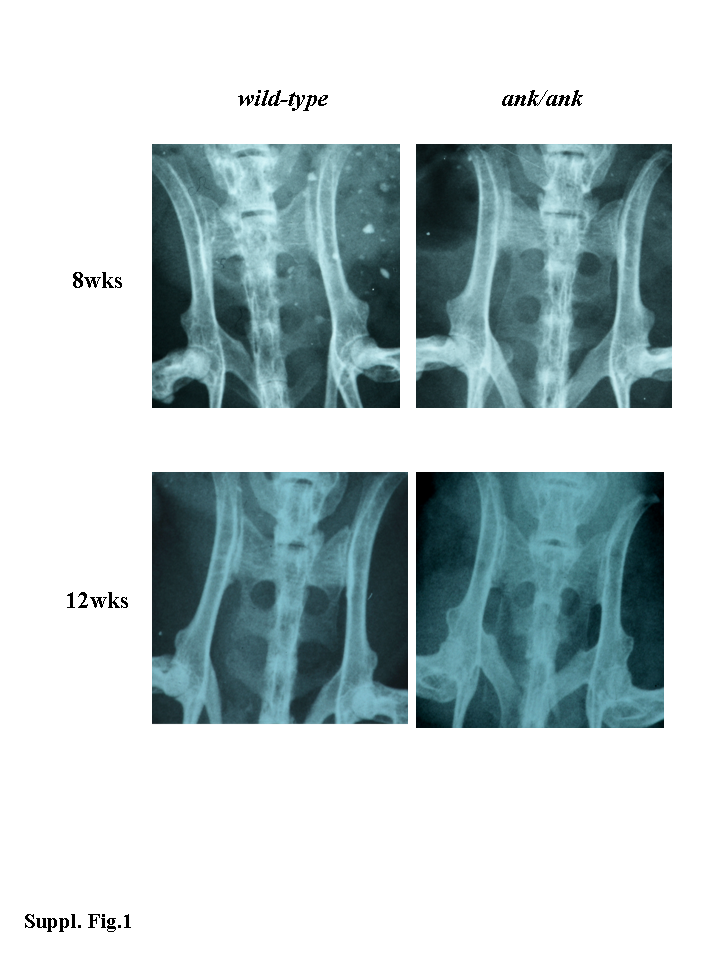

Supplement: Additional file 1 — Radiographs comparing the sacroiliac joints (SIJ) from 8- and 12-week-old wild-type versus ank/ank mice. No ankylosis was detected. [file ar3482-S1.TIFF]

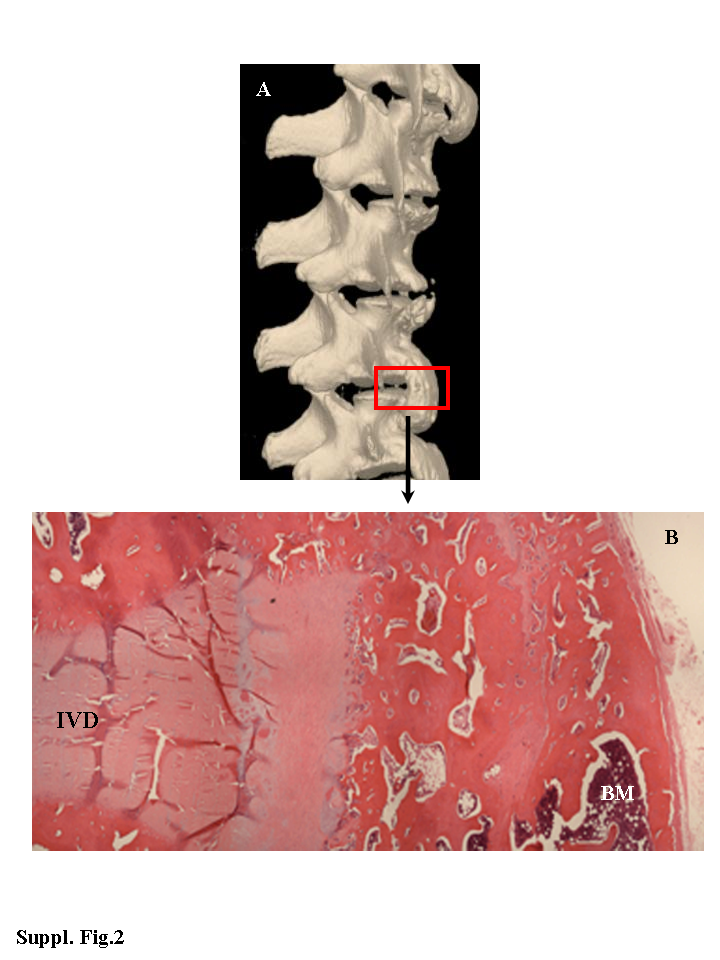

Supplement: Additional file 2 — An old beagle spine with diffuse idiopathic skeletal hyperostosis (DISH). (a) A computed tomography (CT) micrograph showing contiguous bony fusion in the lumbar spine, connecting one vertebra to the next. (b) Histopathology of a typical osteophyte, which contains a contiguous bony fusion mass emanating from the vertebral body, complete with bone marrow (BM). A prominent feature in ank/ank spine (ill-defined connective tissue/fibrous tissue mass) is not observed in the old beagle spine. [file ar3482-S2.TIFF]
